# A feasibility study of the therapeutic application of a mixture of ^67/64^Cu radioisotopes produced by cyclotrons with proton irradiation

**DOI:** 10.1002/mp.15524

**Published:** 2022-02-20

**Authors:** Laura De Nardo, Gaia Pupillo, Liliana Mou, Juan Esposito, Antonio Rosato, Laura Meléndez‐Alafort

**Affiliations:** ^1^ Department of Physics and Astronomy University of Padua Via Marzolo 8 Padova 35131 Italy; ^2^ INFN‐Padova National Institute of Nuclear Physics Via Marzolo 8 Padova 35131 Italy; ^3^ INFN‐Legnaro National Laboratories National Institute of Nuclear Physics Viale dell'Università 2 Legnaro 35020 Italy; ^4^ Department of Surgery Oncology and Gastroenterology University of Padua Padova Italy; ^5^ Veneto Institute of Oncology IOV‐IRCCS Via Gattamelata 64 Padova 35138 Italy

**Keywords:** ^67^CuCl_2_, copper radioisotope mixture, copper radioisotope production, cyclotron physics/radionuclide production, internal dosimetry, radiation dosimetry and risk, theranostic copper radioisotopes

## Abstract

**Purpose:**

^64^Cu and ^67^Cu radioisotopes have nuclear characteristics suitable for nuclear medicine applications. The production of ^64^Cu is already well established. However, the production of ^67^Cu in quantities suitable to conduct clinical trials is more challenging as it leads to the coproduction of other Cu isotopes, in particular ^64^Cu. The aim of this study is to investigate the possibility of using a CuCl_2_ solution with a mixture of ^67/64^Cu radioisotopes for therapeutic purposes, providing an alternative solution for the cyclotron production problem.

**Methods:**

Copper radioisotopes activities were calculated by considering proton beam irradiation of the following targets: (i) ^70^Zn in the energy range 70–45 MeV; (ii) ^68^Zn in the energy range 70–35 MeV; (iii) a combination of ^70^Zn (70–55 MeV) and ^68^Zn (55–35 MeV). The contribution of each copper radioisotope to the human‐absorbed dose was estimated with OLINDA/EXM software using the biokinetic model for CuCl_2_ published by ICRP 53. The total absorbed dose generated by the ^67/64^CuCl_2_ mixture, obtained through different production routes, was calculated at different times after the end of the bombardment (EOB). A simple spherical model was used to simulate tumors of different sizes containing uniformly distributed ^67/64^Cu mixture and to calculate the absorbed dose of self‐irradiation. The biological damage produced by ^67^Cu and ^64^Cu was also evaluated through cellular dosimetry and cell surviving fraction assessment using the MIRDcell code, considering two prostate cancer cell lines with different radiosensitivity.

**Results:**

The absorbed dose to healthy organs and the effective dose (ED) per unit of administered activity of ^67^CuCl_2_ are higher than those of ^64^CuCl_2_. Absorbed dose values per unit of administered activity of ^67/64^CuCl_2_ mixture increase with time after the EOB because the amount of ^67^Cu in the mixture increases. Survival data showed that the biological damage caused per each decay of ^67^Cu is greater than that of ^64^Cu, assuming that radionuclides remain accumulated in the cell cytoplasm. Sphere model calculations demonstrated that ^64^Cu administered activity must be about five times higher than that of ^67^Cu to obtain the same absorbed dose for tumor mass between 0.01 and 10 g and about 10 times higher for very small spheres. Consequently, the ^64^CuCl_2_‐absorbed dose to healthy organs will reach higher values than those of ^67^CuCl_2_. The supplemental activity of the ^67/64^CuCl_2_ mixture, required to get the same tumor‐absorbed dose produced by ^67^CuCl_2_, triggers a dose increment (DI) in healthy organs. The waiting time post‐EOB necessary to keep this DI below 10% (*t*_10%_) depends on the irradiation methods employed for the production of the ^67/64^CuCl_2_ mixture.

**Conclusions:**

A mixture of cyclotron produced ^67/64^Cu radioisotopes proved to be an alternative solution for the therapeutic use of CuCl_2_ with minimal DI to healthy organs compared with pure ^67^Cu. Irradiation of a ^70^Zn+^68^Zn target in the 70–35 MeV proton energy range for 185 h appears to be the best option from among all the production routes investigated, as it gives the maximum amount of activity, the shortest *t*_10%_ (10 h), and less than 1% of ^61^Cu and ^60^Cu impurities.

## 1 INTRODUCTION

Copper is an essential element for a multitude of biological processes, being a catalytic cofactor of many enzymes and a key structural component of functional proteins with fundamental roles in cellular biology.^1^ Copper also plays a key role in cell replication and growth, and it has been found to be deeply involved in cancer development and progression. The potential role of Cu^2+^ ions and their ability to selectively target cancerous cells was recently assessed.^2^ Preliminary results showed a high uptake of ^64^Cu^2+^ in prostate cancer cells, demonstrating the great diagnostic potential of ^64^CuCl_2_ for cancer.^3^ The therapeutic potential of ^64^CuCl_2_ was also assessed in malignant melanoma^4^ and glioblastoma tumor‐bearing mice^5^ and a high tumor uptake of ^67^CuCl_2_ was observed in colorectal tumor‐bearing mice.^6^ Despite only two preliminary reports have demonstrated a therapeutic effect of ^64^CuCl_2_ in patients affected by relapsing malignancies (i.e. glioblastoma, prostate and uterine cancer),^7^, ^8^ these findings suggest that both ^64^CuCl_2_ and ^67^CuCl_2_ could be used to further treat these types of tumors in future.

The five copper radioisotopes with the nuclear characteristics most suitable for nuclear medicine applications are ^60^Cu, ^61^Cu, ^62^Cu, ^64^Cu, and ^67^Cu.^9^ Among them, ^60^Cu (*t*_1/2 _= 23.7 m), ^61^Cu (*t*_1/2 _= 3.333 h), and ^62^Cu (*t*_1/2 _= 9.673 m) are pure positron emitters; ^67^Cu (*t*_1/2 _= 61.83 h) decays emitting a combination of β^–^ particles with *E*_max _= 0.56 MeV (100%) and *γ*‐rays at 92 keV (23%) and 185 keV (48%), suitable for SPECT imaging, and could thus be used as a theranostic agent; ^64^Cu (*t*_1/2 _= 12.7 h) decays mostly through the emission of β^–^ (38%), β^+^ (18%) particles and Auger electrons, so it can find both diagnostic and therapeutic applications. ^64^Cu‐based therapy can be advantageous if the radionuclide is incorporated into the cell nucleus as its Auger electron emission could deliver a very high dose to the DNA, killing the cells.

While ^64^Cu radiopharmaceuticals are employed in the clinical diagnosis of some types of tumors,^10^ the limited availability of ^67^Cu^11^ has to date severely restricted its use, despite its promising results in radioimmunotherapy,^12^, ^13^, ^14^ peptide receptor radionuclide therapy,^15^, ^16^ and PSMA targeting therapy.^17^, ^18^

The production of ^64^Cu is well‐established, and it is mainly based on the use of ^64^Ni or ^68^Zn targets, irradiated by proton or deuteron beams.^19^

The production of ^67^Cu is instead more challenging and still under investigation,^20^ as emerged from the dedicated Coordinated Research Project (CRP) promoted by the International Atomic Energy Agency (IAEA).^21^ It emerges from recent publications on ^67^Cu production^22^, ^23^, ^24^, ^25^ that the use of highly enriched target materials results in a pure final product at the end of irradiation with the ^68^Zn(*γ*,*p*)^67^Cu, ^70^Zn(*p*,*α*)^67^Cu, and ^70^Zn(*d*,*x*)^67^Cu reactions at low energy (*E*_p _< 35 MeV, *E*_d _< 27 MeV). All nuclear reactions concerned have low cross‐section values (below 30 mb), leading to a rather low ^67^Cu yield. In order to increase the proton‐based production of ^67^Cu, it is necessary to use ^68^Zn‐ or ^70^Zn‐enriched targets and irradiations at intermediate beam energies (i.e., larger than 30 MeV). However, this approach leads to the coproduction of Cu isotopic impurities, in particular ^64^Cu. As Cu isotopes cannot be separated by standard (i.e., radiochemical) methods, this is a concern from a pharmaceutical point of view. According to the European Pharmacopeia, the radionuclidic purity of a radiopharmaceutical must indeed be greater than 99%. In general, this limit guarantees that the dose increase due to the impurities remains below 10%.^26^, ^27^ If ^64^Cu is considered an impurity, it will be then necessary a long waiting time after the irradiation of targets to achieve the required radionuclidic purity, losing most of the ^67^Cu produced activity. However, as both ^67^Cu and ^64^Cu have promising therapeutic characteristics, ^64^Cu could not be considered as an impurity, but, on the contrary, as a therapeutic coadjuvant of ^67^Cu, with also the possibility of exploiting its β^+^ emission for the monitoring of the radiopharmaceutical uptake and the biodistribution in the body by PET imaging, with higher accuracy compared to the SPECT imaging allowed by the γ‐emissions of ^67^Cu. Therefore, a combination of the two radionuclides is worthwhile to be investigated.

The energy of released particles is an important parameter to be evaluated for cancer therapy with β emitters because therapeutic effectiveness can be low if electron penetration ranges are greater than the tumor dimensions. Generally, tumors come in a variety of sizes, ranging from a single or a few cells to large tumors with radii of several centimeters. A radionuclide that releases a high absorbed dose to large tumors may be nonoptimal for small ones because a substantial fraction of the β‐particle energy will be delivered to healthy tissues adjacent to the tumors. Therefore, an optimal tumor diameter range for each radionuclide has been identified in order to produce an effective treatment.^28^, ^29^ Wheldon et al.^28^ were the first to propose the use of a panel of β^–^‐emitting radionuclides for clinical scenarios involving a vast number of tumors and metastases of different sizes. The authors reported that the overall level of variation in the probability of cure of tumors with extensive differences in radii could be reduced when using β^–^ emitters with different β end‐point energies.^28^ A clinical study, using a combined ^90^Y/^177^Lu‐DOTATATE therapy, demonstrated that the combination of the two radionuclides with differing β^–^ energy and, therefore, a different maximum range in tissues (2.27 MeV and 10 mm for ^90^Y, and 0.497 MeV and 2–4 mm for ^177^Lu, respectively), produced longer overall patient survival than a single radioisotope treatment.^30^ Nevertheless, it is important to underline that the chemical properties of the same molecule, labeled with different radionuclides, are not identical. The radiolabeled molecules seem to be similar, but can present different stability and biodistribution, because each element has a specific chemical demand arising from its fundamental characteristics such as the atomic number, charge, and radius, which result in a distinct coordination number and geometry.^31^ The advantage of using a radionuclide cocktail with isotopes of the same element is that their labeled conjugates will have the same stability and biodistribution due to identical chemical properties. In case of ^64^Cu and ^67^Cu, despite their different decay schemes, the β^–^ end‐point energies are quite similar (0.65310 and 0.56170 MeV for ^64^Cu and ^67^Cu, respectively). Therefore, a mixture of the two radionuclides is not expected to provide a therapeutic benefit for treating tumors of different sizes, as demonstrated by the similar therapeutic potential of ^64^Cu and ^67^Cu on a per‐decay basis by both in vitro and in vivo studies.^32^, ^33^ However, supposing that the presence of ^64^Cu will not adversely affect the absorbed dose to healthy organs compared with the administration of pure ^67^Cu, the possibility of using a mixture of ^67^Cu and ^64^Cu for therapeutic purposes will provide an alternative solution to the ^67^Cu supply.

This work investigated the production of ^67^Cu/^64^Cu using proton beams up to 70 MeV in three scenarios: (i) the use of ^70^Zn targets in the energy range 70–45 MeV; (ii) the use of ^68^Zn targets in the energy range 70–35 MeV; (iii) the use of a combination of ^70^Zn (70–55 MeV) and ^68^Zn (55–35 MeV) targets, as presented in the INFN patent.^34^

To assess the possibility of using a mixture of ^67/64^Cu radioisotopes for therapeutic purposes, the contribution of each radioisotope to the human‐absorbed dose after the administration of the CuCl_2_ solution was estimated using the biokinetic model published by ICRP 53^35^ with the OLINDA/EXM software's adult male/female reference phantoms.^36^ The total absorbed dose from a CuCl_2_ solution containing a mixture of both radioisotopes was then calculated considering different production methods at different times after the end of bombardment (EOB). Furthermore, a simple model was used to simulate tumors as isolated unit density spheres immersed in an infinite unit density medium and to calculate the absorbed dose attributable to self‐irradiation for the activity uniformly distributed into the spheres. Cellular dosimetry and cell surviving fraction were also evaluated assuming the administration of ^67^CuCl_2_ or ^64^CuCl_2_ to two prostate cancer cell lines with different radiosensitivity to determine the biological damage produced by each radioisotope.

## 2 MATERIALS AND METHODS

### 2.1 Copper‐67 and Copper‐64 production yields

The production of ^67^Cu, ^64^Cu, ^61^Cu, and ^60^Cu radionuclides was calculated with the IAEA tool ISOTOPIA,^37^ taking into account the following priority list for the selection of nuclear cross sections (xs):

- the IAEA recommended values^38^;
- the experimental values available in the literature and the EXFOR database^39^;
- the TALYS estimated trend available in the TENDL library.^40^

These criteria led to the following configuration for the different scenarios: (A) a ^68^Zn target with a proton beam energy in the range 70–35 MeV (the exit energy for a 6.2 mm thick ^68^Zn target): ^67^Cu and ^64^Cu activities were calculated by taking the IAEA xs recommended data into account,^38^ ^61^Cu activity by considering experimental xs values,^39^, ^41^ and ^60^Cu activity considering TENDL nuclear model predictions^40^; (B) a ^70^Zn target with a proton beam in the energy range 70–45 MeV (the exit energy for a 5.08 mm thick ^70^Zn target): ^67^Cu and ^64^Cu activities were calculated by considering experimental xs data,^25^, ^39^ ^61^Cu activity was estimated based on the use of the TENDL library,^40^ and ^60^Cu production was not foreseen; (C) the combined ^70^Zn+^68^Zn target: in the energy range 70–55 MeV (^70^Zn target), ^67^Cu and ^64^Cu activities were calculated by considering experimental data,^25^, ^39^ ^61^Cu activity was based on the use of TENDL‐predicted cross sections,^40^ while ^60^Cu production was not foreseen; in the energy range 35–55 MeV (^68^Zn target), ^67^Cu and ^64^Cu activities were calculated by taking IAEA data into account,^38^ ^61^Cu activity was based on the use of experimental values,^41^ and ^60^Cu activity by considering the TENDL library.^40^

The yield for all the different nuclear reaction routes concerned was estimated by considering a proton beam current of 1 µA and irradiation times of 62 h (corresponding to a saturation factor [SF] of about 50% of ^67^Cu), 124 h (^67^Cu SF ≈ 75%) and 185 h (^67^Cu SF ≈ 88%) as irradiation parameters.

### 2.2 Biokinetic model of CuCl_2_

The biokinetic model published by ICRP 53^35^ was used to estimate the total number of disintegrations in the main human source organs after administration of *^xx^* CuCl_2_.

According to a general first‐order kinetic model, and assuming an immediate uptake into the organs, the fractional activity in a source organ S at time *t*, *A*_s_(*t*), after administration of the activity *A*_0_ is given by the relationship:

(1) $$\frac{A_{S}\left(t\right)}{A_{0}}=F_{S}\;\sum_{i\;=\;1}^{m}a_{i}e^{\left(-\frac{\ln\left(2\right)}{T_{i,\mathrm{eff}}}t\right)}$$

where *F*_s_ is the fractional distribution to organ or tissue S, *m* is the number of elimination components, and *a_i_* is the fraction of *F*_S_ eliminated with effective half‐life *T_i_*_,eff_, which can be calculated from the corresponding biological half‐life *T_i_* and the physical half‐life *T*_p_ of the radioisotope:

(2) $$\frac{1}{T_{i,\mathrm{eff}}}=\frac{1}{T_{p}}\;+\frac{1}{T_{i}}$$

The model parameters to calculate copper uptake in the main human source organs such as the liver, brain, kidneys, pancreas, and in the entire body are reported in Table S1.^35^ The normalized cumulated activity is then calculated according to the formula:

(3) $$\frac{{\tilde{A}}_{S}}{A_{0}}=F_{S}\;\sum_{i\;=\;1}^{m}a_{i}\frac{T_{i,\mathrm{eff}}}{\ln\left(2\right)}$$

### 2.3 Dosimetric calculations applied to human phantoms

Dosimetric calculations for *^xx^* CuCl_2_ were performed with the Organ Level Internal Dose Assessment (OLINDA) software code version 2.2.0,^36^, ^42^ based upon the RADAR method for internal dose estimation,^43^ aiming at obtaining both the absorbed doses per unit of administered activity in each organ and the effective dose (ED). The normalized cumulated activity in the source organs obtained with the ICRP 53 biokinetic model^35^ and both female and male NURBS‐type phantoms,^44^ based on the standardized masses defined by ICRP 89,^45^ were used as input for the calculations with the OLINDA software. Effective dose equivalent (EDE) and ED values were calculated by using the three different tissue‐weighting factors sets, recommended by ICRP 26,^46^ ICRP 60,^47^ and ICRP 103.^48^

Finally, the absorbed doses to different healthy organs (*D*_organ_,_t_) and the total ED (ED_t_) per unit administered activity caused by the mixture of copper radioisotopes obtained from different production methods were calculated at different times after EOB, using the following equations:

(4) $$D_{\mathrm{organ},t}\;\left(t\right)=\sum_{xx}f_{xx_{\mathrm{Cu}}}\left(t\right)\cdot D_{\mathrm{organ},xx_{\mathrm{Cu}}}$$

(5) $$ED_{t}\;\left(t\right)=\sum_{xx}f_{xx_{\mathrm{Cu}}}\left(t\right)\cdot ED_{xx_{\mathrm{Cu}}}$$

where *f_xx_*_Cu_ (*t*) is the fraction of total activity corresponding to *^xx^* Cu radioisotope at the time *t* after EOB and *D*_organ_,*_xx_*_Cu_ and ED*_xx_*_Cu_ are the absorbed dose to an organ and the ED due to unit administered activity of *^XX^* CuCl_2_.

### 2.4 Dosimetric calculations applied to a macroscopic tumor (sphere model)

The OLINDA software's sphere model module was used to simulate tumors as isolated unit density spheres immersed in an infinite unit density medium. This module allows for the evaluation of the absorbed dose solely from self‐irradiation for activity uniformly distributed throughout the spheres. Data are available for discrete sphere masses ranging from 0.01 to 6000 g. Calculations for smaller spheres were performed using the MIRDcell programme,^49^ evaluating self‐doses to spheres ranging from 10 µm of diameter (mass: 5 × 10^–10^ g) up to 2.5 mm (mass: 8 × 10^–3^ g). Both programmes were used to calculate the absorbed doses for ^67^Cu and ^64^Cu radionuclides, which were then compared with the data for ^177^Lu.

The tumor‐absorbed dose generated by the mixture of copper radioisotopes obtained from different irradiations was also calculated at different times after EOB. Calculations were performed assuming an immediate uptake of the ^67/64^CuCl_2_ mixture in the tumor and disregarding biological elimination. The percentage of the number of nuclear transformations (%nt) occurring within the tumor due to each *^xx^* Cu radioisotope in the mixture was evaluated on the basis of the total activity fraction corresponding to each *^xx^* Cu radioisotope at the time of injection and the physical half‐life of the radioisotope:

(6) $$\%nt_{{}^{xx}\mathrm{Cu}}\left(t\right)\;=\;100\cdot \frac{\%A{}^{xx}\mathrm{Cu}\left(t\right)\cdot T_{p}\left({}^{xx}\mathrm{Cu}\right)}{\sum_{xx}\%A{}^{xx}\mathrm{Cu}\left(t\right)\cdot T_{p}\left({}^{xx}\mathrm{Cu}\right)}$$

The tumor‐absorbed dose for the ^67/64^CuCl_2_ mixture was then obtained by weighting the absorbed dose of each *^xx^* Cu radioisotope according to the fraction of decays inside the sphere.

### 2.5 Cellular dosimetry and survival

MIRDcell software^49^ was used to compare the biological damage caused by ^67^Cu or ^64^Cu radionuclides. This programme makes it possible to determine the cellular radiation absorbed doses as well as the surviving fraction of cells in a 3D multicellular cluster after radionuclide treatment. Calculations were performed considering all β and conversion electron emissions with a contribution to the total energy emitted per nuclear transformation greater than 0.1%. Calculations considered the full energy spectrum for β particles. Cellular *S* values (mean absorbed dose per unit cumulated activity in the source region) were obtained using a model that considers the cell as two concentric spheres with a 10 and 4 µm radius, representing the whole cell (c) and its nucleus (n), respectively. The cell size was selected based upon the mean size of some of the most studied cancer cell lines,^50^ whereas the cell nucleus size was calculated by using the assumption that the nucleus volume is approximately 8% of the whole cell volume.^51^ The region between both spheres represents the cytoplasm (cy), whereas the surface of the outer larger sphere represents the cell surface.

The cellular *S* value is a dose factor that is determined by the radioisotope used and the spatial relationship between the target and the source region. In this work, cellular *S* values were obtained assuming that radioactivity was uniformly distributed inside one of the cell regions (source region) and taking into account different distances between the target and the source cells (from 20 to 124 µm). Two types of treatment were studied: the first one assuming that the entire cell was both the source and target region, whereas the second one assuming that the cell nucleus was the target region and the cytoplasm the source region. Finally, calculated *S* values were used to obtain the absorbed dose (*D*) to the target region using the following equation:

(7) $$D_{\mathrm{target}\leftarrow \mathrm{source}\;}=N_{\mathrm{source}\;}\times S_{\mathrm{target}\leftarrow \mathrm{source}}$$

where *N*_source_ is the number of disintegrations in the source region per unit of administered activity (Bq‐h/Bq).

The MIRDcell programme was used to estimate survival for each treatment, assuming a cluster of cells with a spherical shape and a radius of 124 µm, containing 1021 cells with a distance of 20 µm between centers of neighboring cells, and considering that only 50% of the cells were labeled with radioactivity. The programme randomly selects labeled cells in the cluster. Cell activity can vary from zero up to a maximum activity, which in this study was set at 0.02 Bq per cell. The time‐integrated activity coefficient, also known as residence time, representing the cumulative number of nuclear transformations (Bq‐h) occurring in the source region per unit administered activity *A*_0_ (Bq), was set at 100 h for both ^67^Cu and ^64^Cu radionuclides.

The surviving fraction was obtained by using the linear quadratic model, which assumes that each cell is killed due to the inactivation of two or more targets and considers two possibilities: lethal damage when the cell injury is irreparable, and sublethal damage when the injury is reparable by the cell itself. Then cell survival curve can be determined through two components, *αD*, which accounts for the linear behavior (proportional to the radiation dose, related to the irreparable injury), and *βD*,^2^ which is proportional to the square of the radiation dose (related to the repairable damage). Survival curves were obtained taking into account the absorbed dose generated by both the radiation emitted within the same cell (self‐dose [*D*_self_]) and the radiation emitted by neighboring cells (cross dose [*D*_cross_]), using the next equation:

(8) $$P=e^{-\alpha_{\mathrm{self}}D_{\mathrm{self}}-\beta_{\mathrm{self}}{D^{2}}_{\mathrm{self}}}\times \;e^{-\alpha_{\mathrm{cross}}D_{\mathrm{cross}}-\beta_{\mathrm{cross}}{D^{2}}_{\mathrm{cross}}}$$

where *α*_self_ and *β*_self_ and *α*_cross_ and *β*_cross_ are the linear quadratic parameters that characterize the cellular response to *D*_self_ and to *D*_cross_, respectively. Calculations were carried out using *α*_self _= *α*_cross _= *α* and *β*_self _= *β*_cross _= *β* and choosing *α* and *β* values reported for two types of prostate cancer cell lines with different radiosensitivity, LNCaP (*α* = 1.081 and *β* = 0) and PC3 (*α* = 0.551 and *β* = 0.021).^52^

The biological damage caused by Cu‐radionuclides was also compared with that obtained with ^177^Lu, currently the most used radionuclide in theranostics. Therefore, the MIRDcell programme was also run under the same conditions by considering the radionuclide ^177^Lu.

## 3 RESULTS

### 3.1 ^67^Cu and ^64^Cu production yields

The production yields of ^67^Cu, ^64^Cu and the radioisotopic impurities ^61^Cu and ^60^Cu are reported in Table 1. Production yields were estimated considering the proton irradiation of both ^68^Zn and ^70^Zn targets for the different scenarios and irradiation times described.

**TABLE 1:** Calculated yields (MBq/µA) of ^67^Cu,^64^Cu,^61^Cu, and ^60^Cu radionuclides obtained at the EOB through the proton irradiation of ^68^Zn and ^70^Zn targets for the different scenarios and irradiation times, the waiting time necessary to achieve a ^67^Cu radionuclidic purity of 99% and the amount of ^67^Cu activity at this time

	Irr. time (h)	^67^Cu at EOB (MBq/µA)	^64^Cu at EOB (MBq/µA)	^61^Cu at EOB (MBq/µA)	^60^Cu at EOB (MBq/µA)	*t*_99%_ (h)	^67^Cu at t_99%_ (MBq/µA)
^68^Zn: 70–35 MeV	62	1240.1	6512.0	1140.1	26.5	145	244.1
	124	1859.4	6732.9	1140.1	26.5	136	404.8
	185	2165.2	6740.4	1140.1	26.5	133	487.5
^70^ Zn: 70–45 MeV	62	1751.7	7506.7	11.7	–	139	368.7
	124	2626.5	7761.4	11.7	–	131	604.8
	185	3058.5	7770.0	11.7	–	128	728.3
^70^ Zn: 70–55 MeV + ^68^ Zn: 55–35 MeV	62	1881.3	5825.0	40.0	0.0012	132	428.3
	124	2820.9	6022.6	40.0	0.0012	123	710.5
	185	3284.9	6029.3	40.0	0.0012	120	855.6

**FIGURE 1:**
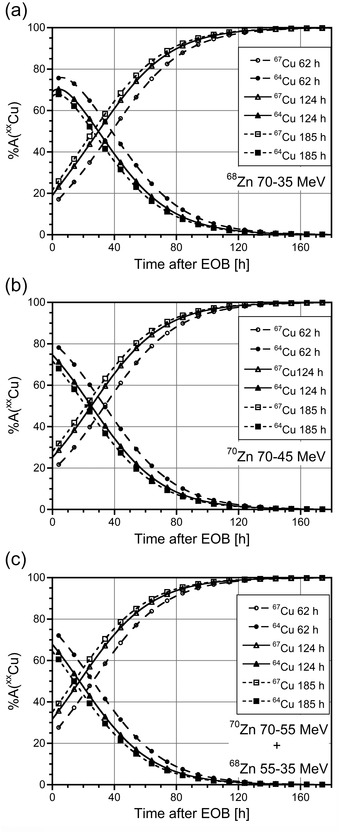
Percentage of activity due to ^67^Cu and ^64^Cu radionuclides as a function of time postirradiation, obtained with a 1 µA proton beam and different irradiation times (circles: 62 h; triangles: 124 h; squares: 185 h) of (a) a ^68^Zn target in the energy range 70–35 MeV; (b) a ^70^Zn target in the energy range 70–45 MeV; (c) a composite ^70^Zn–^68^Zn target in the energy range 70–55 and 55–35 MeV, respectively.

Table 1 demonstrates that both ^67^Cu and ^64^Cu are produced in all the scenarios investigated and their amount increases with the irradiation time. The activity of ^64^Cu is always greater than that of ^67^Cu at the EOB. However, due to the different half‐lives of the two radioisotopes, the percentage amount of ^64^Cu activity in the total decreases with time after irradiation, whereas the percentage amount of ^67^Cu activity increases (see Figure 1). However, as also reported in Table 1, considering ^64^Cu as an impurity (besides ^61^Cu and ^60^Cu) with respect to the ^67^Cu production process, the waiting time necessary to achieve a radionuclidic purity higher than 99% (*t*_99%_) would be quite long (between 120 and 145 h, depending upon the irradiation conditions), causing a decay of about 75–80% of the ^67^Cu produced activity. Both ^61^Cu and ^60^Cu radioisotopic impurities are produced by the irradiation of the ^68^Zn target (for both the 70–35 and 55–35 MeV energy ranges), whereas only ^61^Cu is generated by the irradiation of the ^70^Zn target for both 70–45 and 70–55 MeV. The fraction of total activity due to both ^61^Cu and ^60^Cu radionuclides is, however, lower than 1% at EOB for the irradiation of the ^70^Zn target alone or in combination with the ^68^Zn target. The fraction of total activity due to ^61^Cu plus ^60^Cu radionuclides for the irradiation of the ^68^Zn target at 70–35 MeV is about 12–13%. However, this percentage decreases with time, achieving 1% of total activity from 16 to 17 h after the EOB due to the short half‐lives of both ^61^Cu and ^60^Cu radionuclides.

It should be recalled that the irradiation of ^70^Zn targets at low energy (30–10 MeV range) only produces ^67^Cu, yet the amount of activity obtained is rather low: 258.5 MBq/µA for 62 h of irradiation, 387.6 MBq/µA for 124 h, and 451.4 MBq/µA for 185 h, corresponding to about 15% or 65% of the ^67^Cu activity obtained at EOB or at *t*_99%_, respectively, irradiating ^70^Zn at higher energy (70–45 MeV). For this reason, this scenario was not included in the current work.

### 3.2 Dosimetry of *^XX^* CuCl_2_

Table 2 illustrates the normalized cumulated activity in the main source organs, calculated for the copper radioisotopes ^67^Cu, ^64^Cu, ^61^Cu, and ^60^Cu according to the formula (3). The normalized cumulated activity in the rest of the body corresponds to the difference between the cumulated activity evaluated in the total body and the sum of the cumulated activity recorded in the main source organs.

**TABLE 2:** Normalized cumulated activity calculated for ^67^Cu, ^64^Cu, ^61^Cu, and ^60^Cu according to the ICRP 53 biokinetic model

	$\frac{{\tilde{A}}_{S}}{A_{0}}$ (MBq‐h/MBq)			
Organ	^67^Cu	^64^Cu	^61^Cu	^60^Cu
Brain	7.10	1.74	0.47	0.06
Liver	32.4	9.65	2.91	0.37
Kidneys	0.71	0.17	0.05	<0.01
Pancreas	0.14	0.03	0.01	<0.01
Rest of the body	30.60	5.80	1.30	0.14

These results show that the predominant uptake of CuCl_2_ is in the liver, since this organ is involved in the storage and subsequent redistribution of copper ions to other tissues. Consequently, the hepatobiliary system is the most relevant elimination pathway of excess copper ions from the organism.

Table 3 shows the results of dosimetric calculations performed using both the ICRP 89 male and female phantoms^45^ for ^67^Cu–, ^64^Cu–, ^61^Cu–, and ^60^Cu–Cl_2,_ respectively. ED values were obtained with the more recent tissue‐weighting factors given by ICRP 103.^48^ In addition, EDE and ED values were obtained using the given ICRP 26^46^ and ICRP 60^47^ tissue‐weighting factors in order to compare them to EDE results published by ICRP 53^35^ for a hermaphroditic phantom and to other published data.

**TABLE 3:** Organ doses (mGy/MBq), effective dose equivalent (EDE), and effective doses (ED) due to ^67^Cu–, ^64^Cu–, ^61^Cu–, and ^60^Cu–Cl_2_ for the ICRP 89 male and female phantoms calculated with OLINDA 2.2.0 software

Radioisotope	^67^Cu		^64^Cu		^61^Cu		^60^Cu	
Half‐life	61.83 h		12.72 h		3.333 h		23.7 min	
Target organ	Male	Female	Male	Female	Male	Female	Male	Female
Adrenals	0.148	0.171	0.0522	0.0581	0.0665	0.0729	0.0355	0.0394
Brain	0.483	0.537	0.108	0.12	0.0840	0.0931	0.0327	0.0362
Breasts	–	0.065	–	0.0155	–	0.0143	–	0.00686
Esophagus	0.086	0.107	0.0232	0.0321	0.0258	0.0364	0.0126	0.0178
Eyes	0.059	0.072	0.0133	0.0169	0.0119	0.0154	0.00548	0.00704
Gallbladder wall	0.195	0.157	0.0731	0.0514	0.0949	0.0620	0.0499	0.0309
LLI wall/left colon	0.066	0.077	0.0164	0.0187	0.0161	0.0179	0.00769	0.00835
Small intestine	0.066	0.080	0.0164	0.02	0.0160	0.0198	0.00759	0.00936
stomach wall	0.081	0.091	0.0227	0.0244	0.0247	0.0258	0.0122	0.0125
ULI wall/right colon	0.088	0.095	0.0256	0.0262	0.0286	0.0280	0.0141	0.0139
Rectum	0.053	0.064	0.0111	0.0133	0.00891	0.0106	0.00387	0.00454
Heart wall	0.089	0.089	0.0266	0.0234	0.0300	0.0243	0.0148	0.0116
Kidneys	0.263	0.301	0.0659	0.077	0.0598	0.0714	0.0261	0.0316
Liver	1.780	2.270	0.482	0.612	0.415	0.523	0.168	0.211
Lungs	0.078	0.094	0.0217	0.0261	0.0241	0.0290	0.012	0.0144
Ovaries	–	0.067	–	0.0143	–	0.0119	–	0.00525
Pancreas	0.149	0.206	0.0413	0.0624	0.0420	0.0689	0.0194	0.0332
Prostate	0.054	–	0.0116	–	0.00965	–	0.00427	‐
Salivary glands	0.061	0.070	0.0141	0.0162	0.0128	0.01450	0.00585	0.00662
Red marrow	0.053	0.062	0.0143	0.0166	0.0145	0.0168	0.00701	0.00805
Osteogenic cells	0.080	0.084	0.0137	0.015	0.0125	0.0145	0.00549	0.00648
Spleen	0.062	0.077	0.0152	0.0195	0.0146	0.0191	0.00694	0.00877
Testes	0.047	–	0.00902	–	0.00653	–	0.00265	–
Thymus	0.063	0.076	0.015	0.0177	0.0146	0.0170	0.00694	0.00807
Thyroid	0.055	0.064	0.0121	0.0142	0.0106	0.0122	0.00478	0.00542
Urinary bladder wall	0.052	0.063	0.0107	0.0118	0.00850	0.00911	0.00364	0.00382
Uterus	–	0.066	–	0.0139	–	0.0114	–	0.00499
Total Body	0.101	0.134	0.0231	0.0327	0.0185	0.0286	0.00757	0.0124
EDE (ICRP26) (mSv/MBq)	0.204	0.258	0.0542	0.0677	0.0502	0.0612	0.0220	0.0265
ED (ICRP60) (mSv/MBq)	0.149	0.189	0.0391	0.0497	0.0356	0.0450	0.0155	0.0195
ED (ICRP103) (mSv/MBq)	0.131	0.168	0.0351	0.0444	0.0329	0.0410	0.0146	0.0180

The absorbed doses calculated for ^67^Cu and ^64^Cu radioisotopes with OLINDA 2.2.0 using the male phantom are generally in agreement with values reported by ICRP 53 for the hermaphroditic phantom. The most significant divergences were found for absorbed dose values in the adrenals and in the total body. Higher differences were found for absorbed doses calculated with the female phantom compared with the hermaphroditic one. Consequently, the EDE values calculated for the male phantom (0.204 mSv/MBq for ^67^Cu and 0.0542 mSv/MBq for ^64^Cu) are quite similar to the values published by ICRP 53 (0.22 mSv/MBq for ^67^Cu and 0.053 mSv/MBq for ^64^Cu), whereas EDE values calculated for the female phantom are higher (0.258 mSv/MBq for ^67^Cu and 0.0677 mSv/MBq for ^64^Cu). Comparing the results calculated with OLINDA for both phantoms, it can be observed that the absorbed doses are higher for female than for male phantoms, as already reported for other radiopharmaceuticals.^26^, ^53^, ^54^ In this case, the difference is also due to the fact that the same organ cumulated activities were used for the male and female phantoms. The dosimetric estimation in humans proved that, with both radioisotopes, the liver received the highest dose, followed by the brain and the kidneys. Due to its longer half‐life, the absorbed doses due to ^67^Cu are higher than those due to ^64^Cu by a factor of between 3 and 6, depending upon the organ. This resulted in a 3.8‐fold increased value of ED or EDE, for both female and male phantoms.

As regards the ^61^Cu impurity, it can be observed that despite the almost fourfold shorter half‐life, the absorbed doses and the ED or EDE values due to this radioisotope are quite similar to those due to ^64^Cu. This is a result of the higher total energy emitted by ^61^Cu for nuclear transformation (1.1327 MeV/nt for ^61^Cu and 0.3102 MeV/nt for ^64^Cu^55^). Due to the high energy emitted for nt (4.8087 MeV/nt ^55^), the absorbed doses of ^60^Cu are not negligible, despite its very short half‐life.

### 3.3 Tumor dosimetry (sphere model)

**FIGURE 2:**
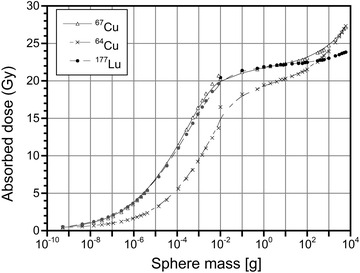
Absorbed dose (Gy) to spheres resulting from a uniform concentration of events (1 decay for µm^3^) due to ^67^Cu, ^64^Cu, and ^177^Lu radioisotopes.

Dose factors obtained with the OLINDA and MIRDcell codes for spheres of mass larger and smaller than 0.01 g, respectively, were used to calculate the absorbed dose to the spheres, considering 1 nt/µm^3^ (that is 10^12^ nt per g of tissue). The results obtained for ^67^Cu, ^64^Cu, and ^177^Lu radionuclides are plotted in Figure 2. It can be noted that the absorbed doses due to ^67^Cu and ^177^Lu are almost identical for small spheres up to 10 g of mass. This is due to the emitted energy per decay in the form of electrons, which is quite similar for the two radionuclides (0.1504 MeV/nt for ^67^Cu and 0.1479 MeV/nt for ^177^Lu). The same holds true for their mean β^–^ energy (0.1359 MeV for ^67^Cu and 0.1333 MeV for ^177^Lu) as reported in Table S2, which describes the main decay characteristics of the ^64^Cu, ^67^Cu, and ^177^Lu radionuclides.^55^ Since a 10 g sphere^56^ absorbs almost all of the energy released by both radionuclides’ electron emission, the absorbed dose for ^67^Cu becomes larger than that for ^177^Lu beyond this size. This is due to the contribution of photons whose emission is higher for ^67^Cu than for ^177^Lu (see Table S2). The lower value of emitted energy per decay in the form of electrons in the case of ^64^Cu (0.1248 MeV/nt) explains the lower absorbed dose values of this radionuclide for the smaller spheres. The ratio of the absorbed dose due to the two copper radionuclides *D*_67Cu_/*D*_64Cu_ is about 1.1 for the 10 g sphere, increasing to 1.2 for the 0.01 g sphere. This value rises strongly as the mass of the spheres decreases, reaching a maximum of about 2.3 for a sphere of 4 × 10^–6^ g (200 µm diameter) as a result of the higher mean energy of electron emission by ^64^Cu compared to that of ^67^Cu (see Table S2). Because of the rather similar total emitted energy per decay (0.2657 MeV/nt for ^67^Cu and 0.3102 MeV/nt for ^64^Cu), the absorbed dose for the two copper radioisotopes only converges for spheres larger than 10^3^ g.

### 3.4 Cellular dosimetry and survival

**FIGURE 3:**
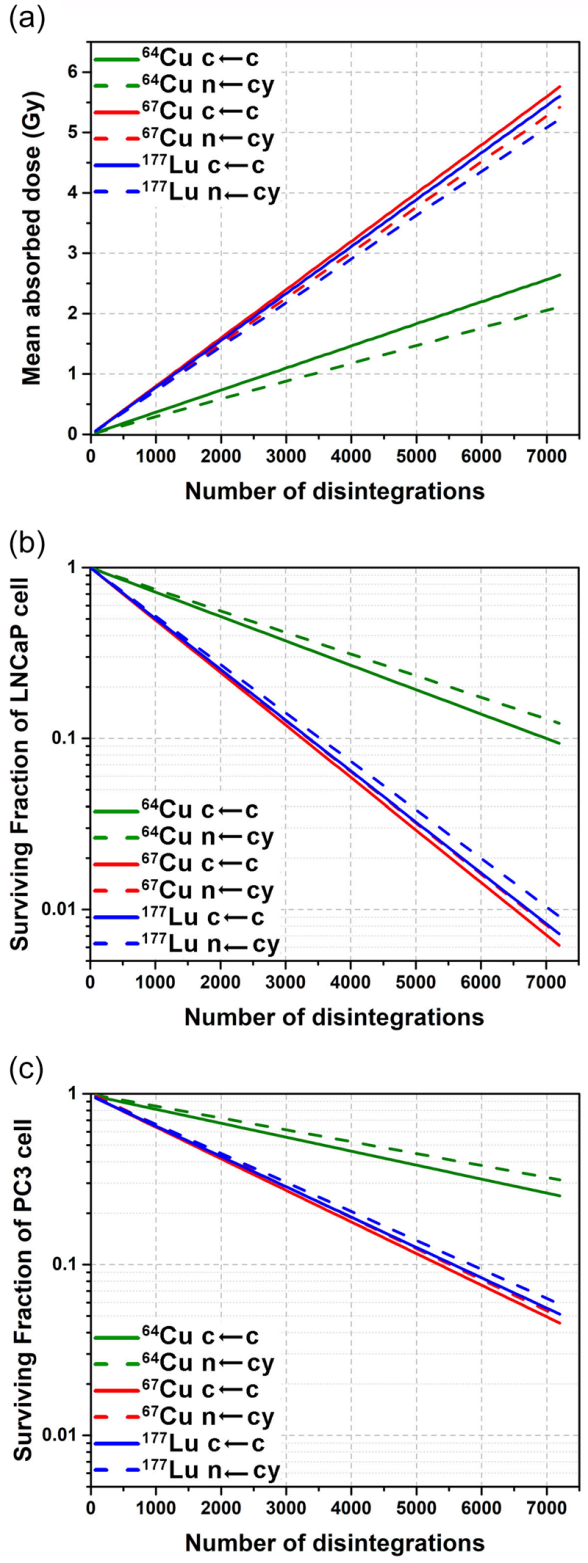
(a) Mean absorbed doses to cells obtained after treatment with ^67^Cu, ^64^Cu, and ^177^Lu and the surviving fractions of (b) LNCaP cells and (c) PC3 cells.

Cellular *S* values calculated for ^67^Cu, ^64^Cu, and ^177^Lu for each target region, nucleus (n), and the whole cell, assuming that the radionuclide was uniformly distributed in one of the source regions, the cytoplasm (cy) or the entire cell (c), demonstrated that in all cases, the self *S* values are the highest. These values decrease as the distance between the source and target cells increases (see Table S3). In general, the calculated ^177^Lu and ^67^Cu *S* values are similar because, as previously discussed, the emitted energy per decay in the form of both radionuclides’ electrons is comparable. Consequently, the mean absorbed doses to cells obtained after treatments with ^177^Lu and ^67^Cu at parity of number of disintegrations were also relatively similar (see Figure 3(a)). As expected, higher differences were found between the mean absorbed doses produced by ^67^Cu and ^64^Cu treatments (see Figure 3(a)).

Mean cell absorbed doses obtained for both LNCaP and PC3 cell lines were the same since only one cell model was used for both of them, however, some differences were found between their surviving fractions (Figures 3(b) and 3(c)). The surviving fraction of LNCaP cells after treatment with ^177^Lu or ^67^Cu was less than 10% and 1% considering 3500 and 7000 disintegrations per cell, respectively. Nevertheless, more than 6000 disintegrations are required to reduce the surviving fraction of the more radioresistant PC3 cells to 10% (see Figure 3(c)). A much larger number of disintegrations is required to achieve the same level of cell survival in the case of ^64^Cu treatments.

### 3.5 Dosimetry of the ^67/64^CuCl_2_ mixture

**FIGURE 4:**
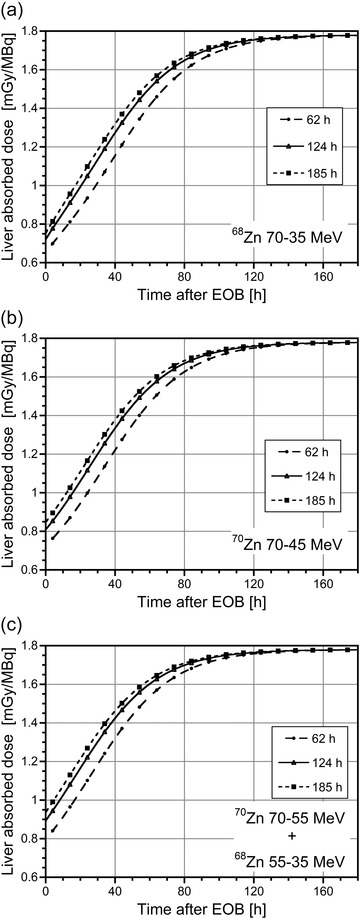
Absorbed dose to the liver per unit of administered activity for the male adult ICRP 89 phantom as a function of time postirradiation due to injection of the ^67/64^CuCl_2_ mixture obtained with 1 µA proton beam and different irradiation times (circles: 62 h; triangles: 124 h; squares: 185 h) of (a) a ^68^Zn target in the energy range 70–35 MeV; (b) a ^70^Zn target in the energy range 70–45 MeV; (c) a composite ^70^Zn–^68^Zn target in the energy range 70–55 and 55–35 MeV, respectively.

The absorbed doses to healthy organs generated by the ^67/64^CuCl_2_ mixture per unit of administered activity were calculated for the male adult ICRP 89 phantom for different production conditions at different times after the EOB. As can be observed in Figure 4, in all cases the absorbed dose to the liver (the most irradiated organ) increases with time. This is due to the increasing contribution of ^67^Cu (see Figure 1) and to its higher value of absorbed dose compared to that of ^64^Cu (see Table 3), approaching the value of 1.78 mGy/MBq, corresponding to 100% ^67^Cu in the mixture.

**FIGURE 5:**
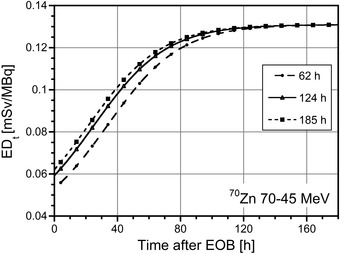
Total ED (ED**_t_**) per unit of administered activity for the male adult ICRP 89 phantom as a function of time postirradiation due to injection of the ^67/64^CuCl_2_ mixture obtained with 1 µA proton beam and different irradiation times (circles: 62 h; triangles: 124 h; squares: 185 h) of a ^70^Zn target in the energy range 70–45 MeV.

The same time dependent behavior was found for the absorbed dose to other healthy organs and also for total ED (ED_t_), as can be observed in Figure 5 for the case of a mixture obtained from ^70^Zn target irradiation in the energy range 70–45 MeV. Similar results were obtained for the irradiation of the ^68^Zn target in the energy range 70–35 MeV and for the composite target ^70^Zn–^68^Zn in the energy range 70–35 MeV. The contribution of the ^61^Cu and ^60^Cu impurities to the liver‐absorbed dose and to the ED_t_ was always less than 10% at the EOB, rapidly decreasing over time.

**FIGURE 6:**
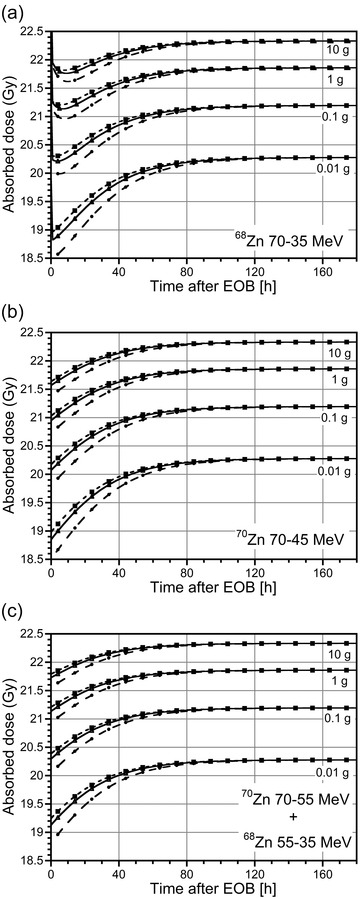
Absorbed doses (Gy) to spheres of different mass (0.01, 0.1, 1, and 10 g) resulting from a uniform concentration of events (1 decay per µm^3^) due to injection of the ^67/64^CuCl_2_ mixture obtained with 1 µA proton beam and different irradiation times (circles: 62 h; triangles: 124 h; squares: 185 h) of (a) a ^68^Zn target in the energy range 70–35 MeV; (b) a ^70^Zn target in the energy range 70–45 MeV; (c) a composite ^70^Zn–^68^Zn target in the energy range 70–55 and 55–35 MeV, respectively.

The tumor‐absorbed dose attributable to the ^67/64^CuCl_2_ mixture, evaluated with the sphere model, was also calculated for different production conditions at different postirradiation times. The results obtained are plotted in Figure 6 for spheres of different mass and a uniform concentration of events (1 decay per µm^3^). The tumor‐absorbed dose increases with time when the ^70^Zn target is irradiated in the energy range 70–45 MeV for each tumor size, reaching a plateau value corresponding to 100% ^67^Cu in the mixture (Figure 6(b)). The absorbed doses are higher for the larger spheres: the absorbed dose for the 10 g sphere is about 15% higher at EOB and 10% higher at the plateau when compared to the 0.01 g sphere (see Figure S1(b)).

The relative increment in the absorbed dose at the plateau with respect to the EOB for 62 h of irradiation (situation corresponding to the largest variation) is less than 10% for the smaller spheres (0.01 and 0.1 g), and about 5% for the larger ones (1 and 10 g) (see Figure S2(b)). Similar results were obtained for the irradiation of the combined ^70^Zn–^68^Zn target (see Figure 6(c), S1(c), and S2(c)). Due to the decay of the ^61^Cu and ^60^Cu impurities, the tumor‐absorbed doses initially decrease with time when a ^68^Zn target is irradiated in the energy range 70–35 MeV; this behavior is more evident in the larger spheres (see Figure 6(a)). After a few hours, the absorbed doses increase again with time until they reach a plateau value, similar to the other irradiation conditions.

## 4 DISCUSSION

The ^64^CuCl_2_ dosimetric evaluation conducted in this study revealed that the liver was the organ that received the highest dose, as already reported in ICRP 53 and validated by other authors in human healthy volunteers,^57^ prostate cancer patients,^3^, ^58^ and glioblastoma multiforme patients.^59^ Table 4 depicts the comparison of our dosimetric data with those previously reported. The liver‐absorbed dose values calculated in this study are higher than those published in the literature, possibly due to the limited number of time points used to estimate radionuclide accumulation in preceding studies. Nevertheless, ED values are in line with those previously published.

**TABLE 4:** Comparison of the liver‐absorbed dose and the effective dose calculated per unit of ^64^CuCl_2_ administered activity in human models

	Human model								
	Capasso et al. 2015	Righi et al. 2018	Panichelli et al. 2016	Avila‐Rodriguez et al. 2017			ICRP 53	This study (ICRP 89 phantom)	
	Male	Male	Male	Male	Female	Mean	Hermaphroditic	Male	Female
Liver (μGy/MBq)	294	271	321	310	421	366	480	482	612
EDE ICRP26 (μSv/MBq)	–	–	40^a^	–	–	–	53	54.2	67.7
ED ICRP60 (μSv/MBq)	33.8	31	40^a^	51.2	61.8	56.5	–	39.1	49.7
ED ICRP103 (μSv/MBq)	–	29.1	–	–	–	–	–	35.1	44.4

^a^ Calculated on the basis of the published organ dose.

Our ^67^CuCl_2_ dosimetric data are also comparable to those reported by ICRP 53, even if higher absorbed doses were calculated for the female phantom compared to the hermaphroditic one used by ICRP 53. It should also be noted that the adoption of the most recent ICRP 103 tissue weighting factors determines a substantial decrease of the ED values, for both male and female phantoms and for both ^64^CuCl_2_ and ^67^CuCl_2_, compared with the EDE values based on the ICRP 26 data set used in ICRP 53 evaluation (see Table 3). The overall consistency of our dosimetric evaluation with published data is encouraging for the application of the same model to the ^67/64^CuCl_2_ mixture.

Absorbed doses to healthy organs per unit of administered activity of ^67^CuCl_2_ are higher by a factor of between 3 and 6 (3.7 for the liver) compared with those attributable to ^64^CuCl_2_, resulting in an ED coefficient that is 3.8 times higher (see Table 3). Nevertheless, given that for most organs the maximum tolerated dose (MTD) to radiation is in the order of some tens of Gy, and the MTD for the gonads and red bone marrow are as low as 1–2 Gy,^60^, ^61^ our dosimetric estimations suggest that it is feasible to administer ^67^CuCl_2_ therapeutic activities in the order of several GBq without jeopardizing the function of these organs. In the case of ^67/64^CuCl_2_, the amount of ^67^Cu in the mixture increases with time after the EOB and, therefore, the absorbed dose to healthy organs and ED values per unit of administered activity increase as well.

Absorbed dose calculations using the sphere model demonstrated that approximately the same total number of ^67^Cu and ^177^Lu radioactive decays are required for the same absorbed dose to a tumor of up to 10 g of mass (see Figure 2). In general, the biodistribution of ^67^Cu‐ and ^177^Lu‐radiopharmaceuticals will be different. However, assuming that the same fraction of administered activities (*A*_0_) accumulates in the tumor for both radionuclides, and considering an immediate uptake without biological elimination, it follows that the same absorbed doses can be attained with ^67^Cu and ^177^Lu by scaling *A*_0_ according to the radioisotope half‐lives (*A*_0 _= nt ln2/*T*_1/2_). Therefore, the required activity of ^67^Cu will be about 2.6 times higher than the activity of ^177^Lu. Given that a 10–20% higher value of radioactive decays is necessary in the case of ^64^Cu compared with ^177^Lu to produce the same absorbed doses for tumor masses ranging between 0.01 and 10 g, the required activity of ^64^Cu will be about 14–15 times higher than that of ^177^Lu. Consequently, when comparing the two copper radioisotopes, the ^64^Cu administered activity must be about 5.5 higher than that of ^67^Cu to get the same tumor‐absorbed dose in this range of sizes, causing the absorbed dose to healthy organs and ED to be higher with respect to ^67^CuCl_2_. The number of ^64^Cu disintegrations necessary to release the same absorbed dose attributed to ^67^Cu becomes about two times higher for very small spheres, necessitating up to 10 times higher ^64^Cu activity in these cases.

However, the biological effect of ^64^Cu would be much higher than that of ^177^Lu or ^67^Cu if this radionuclide were incorporated into the cell nucleus, close to the DNA, because the ^64^Cu Auger electrons would produce high‐density ionizations and high‐energy deposition in a few nanometers. Consequently, the biological effectiveness of Auger electrons emitted inside the cell nucleus could be similar to that of *α* particles, but it would be minimal if the particles were emitted outside the nucleus. Therefore, to calculate the survival fraction of cells after treatment with an Auger‐electron‐emitting radionuclide localized inside the nucleus cell, it is generally necessary to make a distinction between self‐dose and cross‐dose parameters (see Equation 8).^62^ It was discovered that a protein called Atox1 could transport copper into the cell's nucleus,^63^ but it was recently reported that CuCl_2_ could be accumulated inside the nucleus only if it is present in cytotoxic concentrations.^64^ Given that the concentrations of administered radiopharmaceuticals are several orders of magnitude below cytotoxic concentrations, the amount of Cu in the cell's nucleus would be minimal. Consequently, we used the same *α* and *β* values for self‐doses and cross doses to calculate the surviving fraction for all the radionuclides studied.

The evaluation of mean cell absorbed doses and cell survival after both treatments with all radionuclides studied revealed that, when it was assumed that radioactivity was distributed evenly throughout the cell, higher values of absorbed doses were obtained compared to the more realistic approach which considered the cytoplasm as the source region (see Figure 3(a)). The small differences between the mean absorbed doses obtained with both kinds of treatments for ^177^Lu or ^67^Cu do not change the biological effects, since the cell surviving fractions of both treatments are almost identical (see Figures 3(b) and 3(c)). Treatment with ^64^Cu, however, cause lower values of absorbed doses to the cells, producing less biological damage because it was considered that, in these treatment conditions, CuCl_2_ is not concentrated inside the cell's nucleus (see Figures 3(b) and 3(c)). Therefore, in these hypotheses, not only a greater amount of ^64^Cu activity must be injected to obtain the same number of ^67^Cu decays, due to the different radioisotopes half‐lives, but also an additional activity must be administered to obtain the same absorbed dose levels, and consequently the same cell survival. When considering the ^67/64^CuCl_2_ mixture, the supplemental activity necessary to get the same tumor‐absorbed dose produced by ^67^CuCl_2_ depends on the time of administration, since the ^67^Cu concentration in the mixture increases with time after EOB (see Figure 1), as does the relative absorbed dose resulting from a uniform concentration of events, *D*_mix_(*t*)/*D*_67Cu_ (see Figure S2). For example, with *D*_mix_(*t* = 0)/*D*_67Cu_ ≈ 0.9 for the 0.01 g sphere and an irradiation time of 62 h, approximately 10% more decays of the mixture are required at EOB when compared with those of ^67^CuCl_2_ in order to produce the same absorbed dose. The number of decays occurring in the sphere per unit of administered activity of the mixture, nt_mix_/*A*_0,_ are given by the equation:

(9) $$\begin{array}{ccc} \frac{nt_{\mathrm{mix}}\left(t\right)\;}{A_{0}} & = & k\frac{1}{100}\left[\%A^{67}\mathrm{Cu}\left(t\right)\cdot T_{p}\left({}^{67}\mathrm{Cu}\right)\right.\\ & & +\;\left.\%A{}^{64}\mathrm{Cu}\left(t\right)\cdot T_{p}\left({}^{64}\mathrm{Cu}\right)\right] \end{array}$$

where *k* is a proportionality constant, representing the fraction of Cu radioisotopes accumulating inside the tumor.

The percentage of ^64^Cu activity in the mixture obtained by the irradiation of the ^70^Zn target in the energy range 70–45 MeV is about 80% at EOB, giving the coefficient nt_67Cu_/nt_mix_(*t* = 0) = 2.78, which decreases with time after EOB. By considering this coefficient's ratio and the relative absorbed dose attributed to the mixture *D*_mix_(*t*)/*D*_67Cu_, it is possible to calculate the increase in the activity of the ^67/64^CuCl_2_ mixture necessary to obtain the same absorbed dose in the sphere as when using ^67^CuCl_2_:

(10) $$\frac{A_{\mathrm{mix}}\left(t\right)\;}{A_{67\mathrm{Cu}}}=\left(\frac{nt_{67\mathrm{Cu}}\;}{nt_{\mathrm{mix}}\left(t\right)}\right)/\left(\frac{D_{\mathrm{mix}}\left(t\right)}{D_{67\mathrm{Cu}}}\right)$$

This suggests that the administered activity of the ^67/64^CuCl_2_ mixture must be almost three times higher than that of ^67^CuCl_2_ at EOB in order to obtain an equivalent absorbed dose to the 0.01 g sphere.

The dose increment (DI) caused by the use of the ^67/64^CuCl_2_ mixture rather than ^67^CuCl_2_ can be estimated by multiplying the *A*_mix_/*A*_67Cu_ coefficient for the liver‐absorbed dose per unit of administered activity or the ED_t_ value per unit of administered activity (see Figures 4 and 5). For the considered scenario, the increase in the liver‐absorbed dose and in the ED is about 25% at EOB, decreasing to almost 10% approximately 30 h after EOB. Setting the maximum DI limit to 10% after administering the ^67/64^CuCl_2_ mixture, the waiting time required to reach this limit (*t*_10%_) after the EOB can be used to compare the quality of the different ^67/64^CuCl_2_ mixtures_._ Table 5 shows the values of *t*_10%_ and the total activity available at that time, evaluated for the different scenarios and taking the sphere of 0.01 g of mass as a reference. For all the different scenarios, the percentage of ^67^Cu activity at *t*_10%_ is about 45% and the *A*_mix_/*A*_67Cu_ coefficient at this time is about 1.8. As irradiation time rises, the amount of available total activity increases and the *t*_10%_ decreases in all cases (see Table 5). A comparison of the amount of activity of ^67/64^CuCl_2_ available at *t*_10%_ with that of ^67^CuCl_2_ at *t*_99%_, reported in Table 1, clearly indicates the advantage of administering the radionuclidic mix instead of the pure ^67^Cu radioisotope, even taking into account that a greater amount of mixing activity is required. It should be noted that the estimated production yields of all the radionuclides of interest are based on the hypothesis of 100% isotopically enriched target material. However, the material available on the international market for use as a target may have a lower enrichment level (materials with enrichment levels higher than 98.7% for ^70^ZnO and 99% for ^68^ZnO are currently available) and different amounts of Cu isotopes will be produced based on the specific target composition. Given that the natural abundance of ^70^Zn is only 0.61% and that of ^68^Zn is 18.45%,^65^ the price of these enriched materials varies, with the price of ^70^Zn approximately four times more expensive than that of ^68^Zn. From a technical point of view, it is customary to recover and reuse costly enriched materials in the routine production of radionuclides.^19^

**TABLE 5:** Minimum waiting time necessary after EOB to keep the dose increment lower than 10% (*t*_10%_) and the activity (MBq/µA) of the ^67^Cu and ^67^Cu + ^64^Cu mixture at that time obtained through the proton irradiation of ^68^Zn and ^70^Zn targets for different scenarios and irradiation times

	Irr. time (h)	*t*_10%_(h)	^67^Cu + ^64^Cu(MBq/µA)	^67^Cu(MBq/µA)
^68^Zn 70–35 MeV	62	35	1801.8	837.6
	124	26	3018.5	1389.3
	185	23	3594.2	1673.1
^70^Zn 70–45 MeV	62	30	2711.6	1251.4
	124	21	4542.8	2075.6
	185	18	5409.0	2499.6
^70^Zn 70–55 MeV + ^68^Zn 55–35 MeV	62	22	3223.4	1470.1
	124	13	5400.9	2438.3
	185	10	6430.0	2936.5

## 5 CONCLUSIONS

This study assessed the feasibility of using a ^67/64^Cu radioisotope mixture for therapeutic purposes by calculating the total absorbed dose into unit density spheres through the simulation of small‐sized tumors after administration of a ^67/64^CuCl_2_ solution. Owing to the increased contribution of ^67^Cu in the mixture, it was found that the DI resulting from the administration of the ^67/64^CuCl_2_ mixture rather than ^67^CuCl_2_ decreases with time after EOB. The post‐EOB waiting time required to reduce this increment to below 10% (*t*_10%_) depends upon the choice of target and irradiation conditions. The irradiation of a multilayer target composed of ^70^Zn+^68^Zn for 185 h appears to be the best option for CuCl_2_ administration from among all the production parameters studied, since maximum activity was obtained under this condition with the shortest *t*_10%_ (10 h) and less than 1% calculated percentages of ^61^Cu and ^60^Cu impurities. Based on these results, we can conclude that the use of a ^67/64^Cu mixture for therapy could be an advantage because the larger amount of available activity will allow to treat more patients and to reduce the cost of the treatment.

## FUNDING

This work was supported by the COME experiment (funded by INFN) as part of the activities of the LARAMED project of the INFN‐LNL and it was also included in the framework of the IAEA Coordinated Research Project (CRP) on “Therapeutic Radiopharmaceuticals Labelled with New Emerging Radionuclides (67Cu, 186Re, 47Sc)” (IAEA CRP No. F22053).

## CONFLICT OF INTEREST

The authors have no conflicts of interest to disclose.

## Supporting information



Supporting information
