# Supplementary material for: A feasibility study of the therapeutic application of a mixture of 67/64Cu radioisotopes produced by cyclotrons with proton irradiation
Source: Med Phys. 2022 Feb 20;49(4):2709–24. doi: 10.1002/mp.15524 (PMC9305914; doi:10.1002/mp.15524)
Supplement: Supplementary file 1 — Supporting information [file MP-49-2709-s001.docx]

**A feasibility study of the Therapeutic Application of a Mixture of ^67/64^Cu Radioisotopes produced by cyclotrons with proton irradiation**

**Supplemental data**

**Table S-1**. Parameters of the ICRU 53 biokinetic model used to calculate CuCl_2_ organ uptake.

| **Organ** | **F_s_** | **T_i_ [days]** | **a_i_** |
| --- | --- | --- | --- |
| Total body | 1.0 | 10 | 1.0 |
| Brain | 0.1 | 10 | 1.0 |
| Liver | 0.65 | 0.5 | 0.15 |
|  |  | 1.5 | 0.33 |
|  |  | 10 | 0.52 |
| Kidneys | 0.01 | 10 | 1.0 |
| Pancreas | 0.002 | 10 | 1.0 |

**Table S-2***.* Main decay characteristics of the radionuclides ^64^Cu, ^67^Cu, and ^177^Lu.

| Radioisotope | ^64^Cu | | | ^67^Cu | | | ^177^Lu | | |
| --- | --- | --- | --- | --- | --- | --- | --- | --- | --- |
| Half-life | 12.700 h | | | 61.83 h | | | 159.528 h | | |
| Decay mode | EC, β^+^, β^-^ | | | β^-^ | | | β^-^ | | |
| Radiations | Yield  (/nt) | Energy (MeV/nt) | Mean energy (MeV) | Yield  (/nt) | Energy (MeV/nt) | Mean energy (MeV) | Yield  (/nt) | Energy (MeV/nt) | Mean energy (MeV) |
| Gamma rays | 4.734∙10^-3^ | 6.37∙10^-3^ | 1.346 | 7.293∙10^-1^ | 1.148∙10^-1^ | 1.574∙10^-1^ | 1.803∙10^-1^ | 3.156∙10^-2^ | 1.750∙10^-1^ |
| X rays | 2.403 | 1.202∙10^-3^ | 5.001∙10^-4^ | 7.759∙10^-1^ | 5.553∙10^-4^ | 7.157∙10^-4^ | 1.374 | 3.540∙10^-3^ | 2.576∙10^-3^ |
| Annh. Photons | 3.482∙10^-1^ | 1.779∙10^-1^ | 5.11∙10^-1^ | - | - | - | - |  |  |
| Tot photons |  | 1.855∙10^-1^ |  |  | 1.154∙10^-1^ |  |  | 3.51∙10^-2^ |  |
| β^+^ | 1.741∙10^-1^ | 4.843∙10^-2^ | 2.782∙10^-1^ | - | - | - |  |  |  |
| β^-^ | 3.900∙10^-1^ | 7.427∙10^-2^ | 1.904∙10^-1^ | 1.00 | 1.359∙10^-1^ | 1.359∙10^-1^ | 1.00 | 1.333∙10^-1^ | 1.333∙10^-1^ |
| IC electrons | 5.777∙10^-7^ | 7.73∙10^-7^ | 1.338 | 1.528∙10^-1^ | 1.374∙10^-2^ | 8.995∙10^-2^ | 1.548∙10^-1^ | 1.352∙10^-2^ | 8.737∙10^-2^ |
| Auger electrons | 1.807 | 2.05∙10^-3^ | 1.134∙10^-3^ | 5.588∙10^-1^ | 7.510∙10^-4^ | 1.344∙10^-3^ | 1.117 | 1.132∙10^-3^ | 1.014∙10^-3^ |
| Tot electrons |  | 1.248∙10^-1^ |  |  | 1.504∙10^-1^ |  |  | 1.479∙10^-1^ |  |

**Table S-3.** S-values calculated for different radionuclides using MIRDcell program and considering different distances between the target and the source cells.

| **Distance**  **(µm)** | **^64^Cu** | | **^67^Cu** | | **^177^Lu** | |
| --- | --- | --- | --- | --- | --- | --- |
|  | **S(c ⃪ c)**  **(Gy/Bq^.^s)** | **S(n ⃪ cy)**  **(Gy/Bq^.^s)** | **S(c ⃪ c)**  **(Gy/Bq^.^s)** | **S(n ⃪ cy)**  **(Gy/Bq^.^s)** | **S(c ⃪ c)**  **(Gy/Bq^.^s)** | **S(n ⃪ cy)**  **(Gy/Bq^.^s)** |
| Self-S | 1.34E-04 | 6.39E-05 | 1.87E-04 | 1.52E-04 | 2.11E-04 | 1.64E-04 |
| 20 | 6.71E-06 | 6.19E-06 | 1.78E-05 | 1.64E-05 | 1.90E-05 | 1.74E-05 |
| 25 | 3.93E-06 | 3.78E-06 | 1.04E-05 | 9.94E-06 | 1.11E-05 | 1.06E-05 |
| 30 | 2.62E-06 | 2.56E-06 | 6.85E-06 | 6.69E-06 | 7.44E-06 | 7.30E-06 |
| 35 | 1.87E-06 | 1.84E-06 | 4.88E-06 | 4.80E-06 | 5.32E-06 | 5.31E-06 |
| 40 | 1.41E-06 | 1.39E-06 | 3.65E-06 | 3.60E-06 | 3.86E-06 | 3.84E-06 |
| 45 | 1.09E-06 | 1.08E-06 | 2.82E-06 | 2.80E-06 | 2.80E-06 | 2.75E-06 |
| 50 | 8.70E-07 | 8.64E-07 | 2.25E-06 | 2.23E-06 | 2.08E-06 | 2.01E-06 |
| 55 | 7.09E-07 | 7.05E-07 | 1.83E-06 | 1.82E-06 | 1.62E-06 | 1.60E-06 |
| 60 | 5.88E-07 | 5.85E-07 | 1.53E-06 | 1.52E-06 | 1.32E-06 | 1.32E-06 |
| 65 | 4.95E-07 | 4.93E-07 | 1.29E-06 | 1.28E-06 | 1.10E-06 | 1.10E-06 |
| 70 | 4.22E-07 | 4.21E-07 | 1.11E-06 | 1.10E-06 | 9.33E-07 | 9.29E-07 |
| 75 | 3.64E-07 | 3.62E-07 | 9.66E-07 | 9.61E-07 | 7.98E-07 | 7.93E-07 |
| 80 | 3.17E-07 | 3.16E-07 | 8.54E-07 | 8.52E-07 | 6.89E-07 | 6.88E-07 |
| 85 | 2.78E-07 | 2.76E-07 | 7.69E-07 | 7.62E-07 | 6.01E-07 | 5.97E-07 |
| 90 | 2.45E-07 | 2.45E-07 | 7.09E-07 | 7.03E-07 | 5.27E-07 | 5.28E-07 |
| 95 | 2.18E-07 | 2.17E-07 | 6.68E-07 | 6.70E-07 | 4.67E-07 | 4.64E-07 |
| 100 | 1.95E-07 | 1.95E-07 | 6.11E-07 | 6.36E-07 | 4.16E-07 | 4.17E-07 |
| 105 | 1.75E-07 | 1.74E-07 | 5.17E-07 | 5.26E-07 | 3.73E-07 | 3.72E-07 |
| 110 | 1.58E-07 | 1.58E-07 | 4.03E-07 | 3.99E-07 | 3.37E-07 | 3.36E-07 |
| 115 | 1.42E-07 | 1.42E-07 | 3.12E-07 | 2.94E-07 | 3.05E-07 | 3.05E-07 |
| 120 | 1.29E-07 | 1.28E-07 | 2.58E-07 | 2.55E-07 | 2.77E-07 | 2.75E-07 |
| 124 | 1.20E-07 | 1.19E-07 | 2.32E-07 | 2.32E-07 | 2.59E-07 | 2.57E-07 |


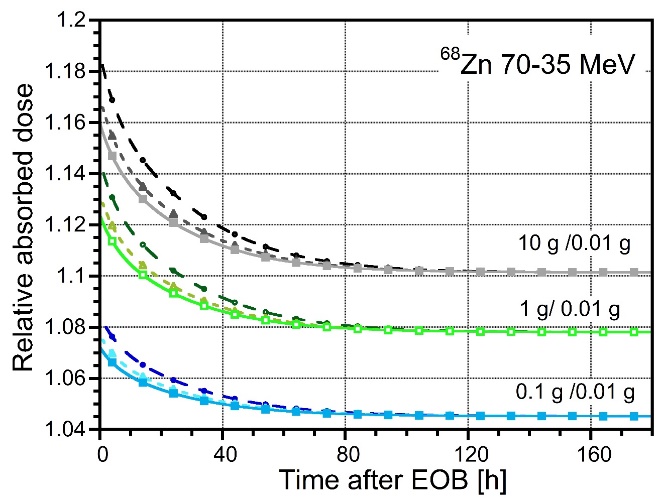
**A)**


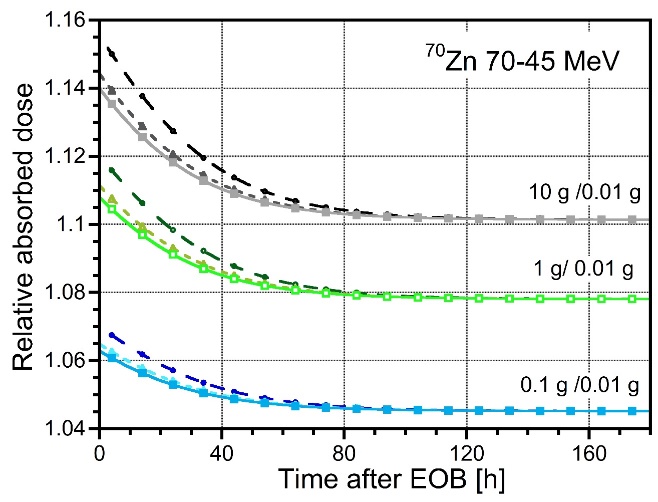
**B)**

**
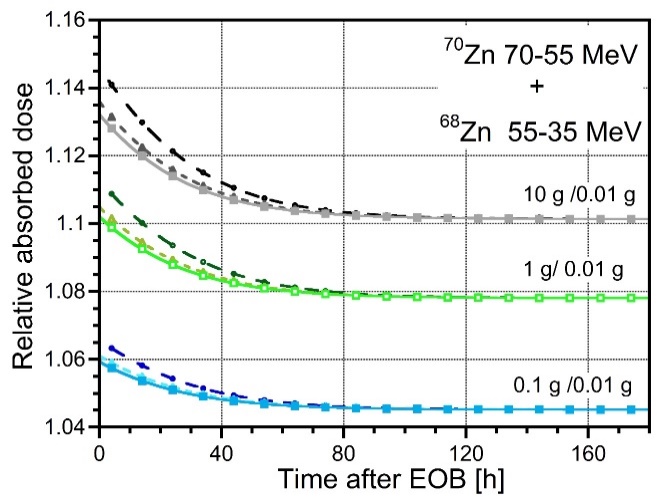
C)**

**Figure S-1**. Relative absorbed dose to spheres of different mass (0.1 g, 1 g and 10 g) compared to that of 0.01 g, resulting from a uniform concentration of events (1 decay for μm^3^) and injection of the ^67/64^CuCl_2_ mixture obtained with 1 μA proton beam and different irradiation times (circles: 62 h; triangles: 124 h; squares: 185 h) of A) a ^68^Zn target in the energy range 70-35 MeV; B) a ^70^Zn target in the energy range 70-45 MeV; C) a composite ^70^Zn-^68^Zn target in the energy range 70-55 MeV and 55-35 MeV, respectively.


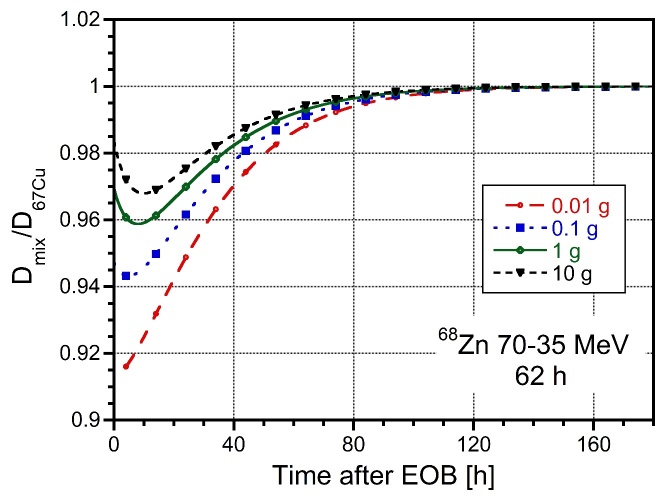
**A)**


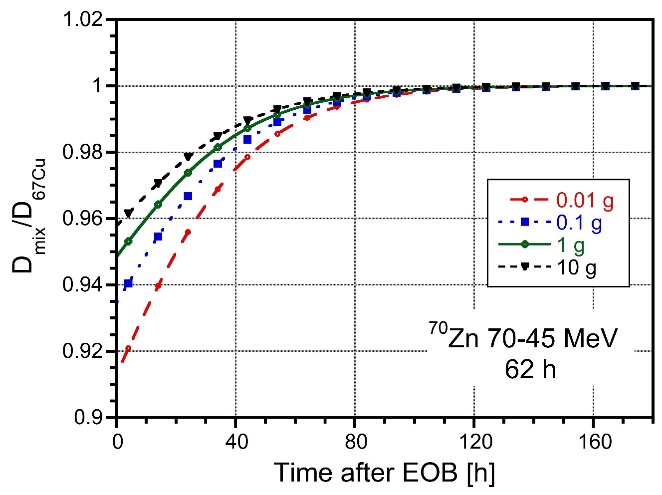
**B)**


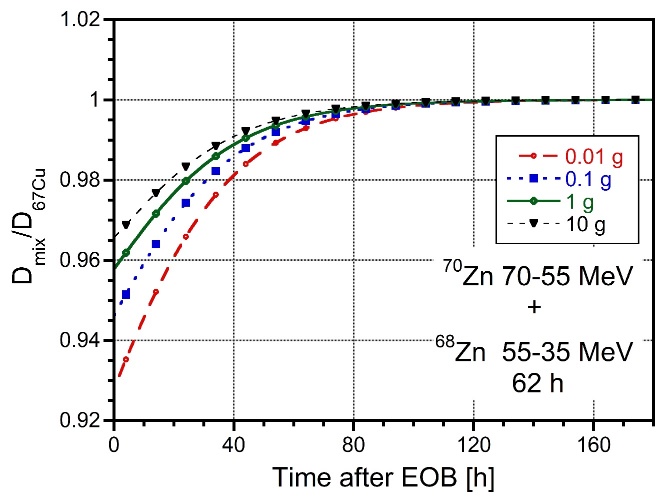
**C)**

**Figure S-2**. Relative absorbed dose (D_mix_/D_67Cu_) to spheres of different mass (0.01 g, 0.1 g, 1 g and 10 g), resulting from a uniform concentration of events (1 decay for μm^3^) and injection of the ^67/64^CuCl_2_ mixture obtained with 1 μA proton beam and 62 h of irradiation of A) a ^68^Zn target in the energy range 70-35 MeV; B) a ^70^Zn target in the energy range 70-45 MeV; C) a composite ^70^Zn-^68^Zn target in the energy range 70-55 MeV and 55-35 MeV, respectively.
